# Draft Genome Sequence of Marine-Derived *Aeromonas caviae* CHZ306, a Potential Chitinase Producer Strain

**DOI:** 10.1128/genomeA.01293-16

**Published:** 2016-11-17

**Authors:** Flávio Augusto Cardozo, Cristina Kraemer Zimpel, Ana Marcia Sa Guimaraes, Adalberto Pessoa, Irma Nelly Gutierrez Rivera

**Affiliations:** a Department of Microbiology, Biomedical Sciences Institute, University of São Paulo, São Paulo, Brazil; b Department of Preventive Veterinary Medicine and Animal Health, University of São Paulo, São Paulo, Brazil; c Department of Biochemical and Pharmaceutical Technology, School of Pharmaceutical Sciences, University of São Paulo, São Paulo, Brazil

## Abstract

We report here a draft genome sequence of *Aeromonas caviae* CHZ306, a marine-derived bacterium with the ability to hydrolyze chitin and express high levels of chitinases. The assembly resulted in 65 scaffolds with approximately 4.78 Mb. Genomic analysis revealed different genes encoding chitin-degrading enzymes that can be used for chitin derivative production.

## GENOME ANNOUNCEMENT

Chitin is the most abundant biopolymer in the marine environment and the main component of the exoskeleton of arthropods, cell walls of fungi, and algae (1). Chitin derivatives (chitosan, chitooligosaccharides and *N*-acetyl-glucosamine) are biocompatible, biodegradable, and show great potential for application in the cosmetic, pharmaceutical, and biomaterial areas (2). These compounds have been traditionally produced by the acid hydrolysis of chitin, but the processes are environmentally harmful and have low yield and high cost (3). The enzymatic hydrolysis of chitin is a sustainable alternative to the chemical process and would not require the use of toxic compounds or generate excessive amounts of wastewater (2).

In order to better understand the chitinase diversity and select specific enzymes for chitin derivative production, this study aimed to sequence and annotate the genome of *Aeromonas caviae* CHZ306, a marine-derived bacterium capable of hydrolyzing chitin and expressing high levels of chitinases.

*A. caviae* CHZ306 was isolated from seawater samples collected in the coast of São Paulo state, Brazil. Genomic DNA from bacterial culture was extracted using the Wizard genomic DNA purification kit (Promega Co., Madison, WI, USA) and quantified using the Qubit fluorometer (Life Technologies, Carlsbad, CA, USA). Paired-end and mate-pair libraries were prepared with Nextera XT DNA library preparation kit (Illumina, San Diego, CA, USA) and Nextera mate-pair library preparation kit (Illumina), respectively, and sequenced in MiSeq platform (Illumina), using the 600-cycle MiSeq reagent kit version 3 (Illumina). *De novo* genome assembly was carried out using the pipeline A5-MiSeq version 20150522 (4), and first-pass annotation was obtained using the NCBI Prokaryotic Genome Annotation Pipeline.

A total of 22,918,174 reads were assembled into 65 scaffolds, resulting in a genome size of ~4.78 Mb, with a G+C content of 60.8%. Annotation resulted in a total of 4,329 coding sequences (CDSs) and 150 RNAs (32 rRNAs, 113 tRNAs, and five noncoding RNAs [ncRNAs]). As some strains of *A. caviae* have been reported to produce systemic infections in humans (5, 6), we also searched for genes involved in the production of major virulence factors of *Aeromonas* species (7–9). Genomic analysis revealed that *A. caviae* CHZ306 contains different genes encoding chitin-degrading enzymes (e.g., chitinase, chitobiase, and beta-*N*-acetylhexosaminidase) of biotechnological interest and virulence factors (e.g., hemolysins, secretion systems, and RTX and zonula occludens toxins) of clinical importance.

### Accession number(s).

The draft genome sequence of *A. caviae* CHZ306 has been deposited at DDBJ/ENA/GenBank under the accession number MDSC00000000. The version described in this paper is the first version, MDSC01000000.

## References

[1] Zobell CE, Rittenberg SC 1938 The occurrence and characteristics of chitinoclastics bacteria in the sea. J Bacteriol 35:75–287. 10.1128/jb.35.3.275-287.1938 PMC545278 16560102

[2] Jung WJ, Park RD 2014 Bioproduction of chitooligosaccharides: present and perspectives. Mar Drugs 12:5328–5356. doi:10.3390/md12115328. 25353253 PMC4245534

[3] Aam BB, Heggset EB, Norberg AL, Sørlie M, Vårum KM, Eijsink VG 2010 Production of chitooligosaccharides and their potential applications in medicine. Mar Drugs 8:1482–1517. doi:10.3390/md8051482. 20559485 PMC2885077

[4] Coil D, Jospin G, Darling AE 2015 A5-miseq: an updated pipeline to assemble microbial genomes from Illumina MiSeq data. Bioinformatics 31:587–589. doi:10.1093/bioinformatics/btu661. 25338718

[5] Janda JM, Guthertz LS, Kokka RP, Shimada T 1994 *Aeromonas* species in septicemia: laboratory characteristics and clinical observations. Clin Infect Dis 19:77–83. doi:10.1093/clinids/19.1.77. 7948561

[6] Janda JM, Abbott SL 1998 Evolving concepts regarding the genus *Aeromonas*: an expanding panorama of species, disease presentation, and unanswered questions. Clin Infect Dis 27:332–344. doi:10.1086/514652. 9709884

[7] Chacón MR, Figueras MJ, Castro-Escarpulli G, Soler L, Guarro J 2003 Distribution of virulence genes in clinical and environmental isolates of *Aeromonas* spp. Antonie Van Leeuwenhoek 84:269–278. doi:10.1023/A:1026042125243. 14574104

[8] Sen K, Rodgers M 2004 Distribution of six virulence factors in *Aeromonas* species isolated from U.S. drinking water utilities: a PCR identification. J Appl Microbiol 97:1077–1086. doi:10.1111/j.1365-2672.2004.02398.x. 15479425

[9] Aguilera-Arreola MG, Hernández-Rodríguez C, Zúñiga G, Figueras MJ, Castro-Escarpulli G 2005 *Aeromonas hydrophila* clinical and environmental ecotypes as revealed by genetic diversity and virulence genes. FEMS Microbiol Lett 242:231–240. doi:10.1016/j.femsle.2004.11.011. 15621443

